# *NiftyFit*: a Software Package for Multi-parametric Model-Fitting of 4D Magnetic Resonance Imaging Data

**DOI:** 10.1007/s12021-016-9297-6

**Published:** 2016-03-14

**Authors:** Andrew Melbourne, Nicolas Toussaint, David Owen, Ivor Simpson, Thanasis Anthopoulos, Enrico De Vita, David Atkinson, Sebastien Ourselin

**Affiliations:** Centre for Medical Image Computing, University College London, London, UK; Academic Neuroradiological Unit, UCL Institute of Neurology, London, UK; Medical Physics, University College Hospital, London, UK

**Keywords:** MRI, Relaxometry, Diffusion, Cerebral blood flow, g-ratio

## Abstract

Multi-modal, multi-parametric Magnetic Resonance (MR) Imaging is becoming an increasingly sophisticated tool for neuroimaging. The relationships between parameters estimated from different individual MR modalities have the potential to transform our understanding of brain function, structure, development and disease. This article describes a new software package for such multi-contrast Magnetic Resonance Imaging that provides a unified model-fitting framework. We describe model-fitting functionality for Arterial Spin Labeled MRI, T1 Relaxometry, T2 relaxometry and Diffusion Weighted imaging, providing command line documentation to generate the figures in the manuscript. Software and data (using the nifti file format) used in this article are simultaneously provided for download*.* We also present some extended applications of the joint model fitting framework applied to diffusion weighted imaging and T2 relaxometry, in order to both improve parameter estimation in these models and generate new parameters that link different MR modalities. *NiftyFit* is intended as a clear and open-source educational release so that the user may adapt and develop their own functionality as they require.

## Introduction

The growth of multi-modal medical imaging datasets, particularly those acquired using MRI has great potential for the development of multi-modality derived imaging biomarkers that combine and summarize two or more types of imaging data. Recent examples are the combination of diffusion weighted MRI (DWI) and Arterial Spin labeled MRI (ASL) as in (Hales and Clark 2013; He et al. 2014; Melbourne et al. 2015), DWI and MR bound pool measurement as in (Stikov et al. 2011; Melbourne et al. 2014a) and DWI and Dynamic Contrast Enhanced MRI (DCE) as in (Hamy et al. 2014). Similarly, familiarity with model-fitting allows bespoke acquisitions to be used to assess quantitative imaging parameters in novel ways (Deoni et al. 2008; Draganski et al. 2011, Vos et al. 2015). Each of these methods allows a measurement and investigation of a tissue property that was not possible until this combination was attempted and allows a unified biological model to be applied. The motivation for the development of accurate imaging biomarkers is three-fold: to improve sensitivity and specificity in individual diagnosis; assess the efficacy of disease modifying therapies in treatment development and to understand the basic science of normal development, disease and ageing. To achieve these goals, it is vital to support the accurate quantification of imaging biomarkers and to facilitate their future development by providing lightweight, easy-to-use software free of cumbersome dependencies and license conflicts. Software of this type should be easy to use on both the individual case and when applied to large datasets and provide a base for future independent development so that the research community can rapidly trial new ideas. A prerequisite for this is that the software be open-source and freely-available, making use of a unified set of common optimization routines. The software package described in this work, termed *NiftyFit* has been developed to serve this purpose. Multi-modal test data is also included as part of the package to provide a base for future research developments and the figures used in this work can be generated from this open-source software and data. Software packages for image analysis exist for multi-purpose image analysis tasks, such as the FSL package that includes tools for registration, segmentation and diffusion imaging (Jenkinson et al. 2012) and the SPM software for volumetric statistical analysis (Friston et al. 2007). Other specialist packages also exist for instance for registration (NiftyReg, Modat et al. 2010) and image segmentation (NiftySeg, Cardoso et al. 2015) but are less common for bespoke multi-contrast parametric designs.

The rest of this paper proceeds as follows: “Materials & Methods” section provides a description of the source code and the image data that forms part of *NiftyFit.* This includes a brief description of the core algorithms used in this work and installation instructions. “Example Applications and Case Studies” section describes how these algorithms are applied to the five imaging modalities presented in this work: arterial spin labeled MRI, T1 and T2 relaxometry, diffusion MRI and their extensions. “Discussion and Future Developments” section finalises the paper with a brief discussion of future work.

## Materials & Methods

### Data Overview

*NiftyFit* includes a dataset consisting of imaging data from 9 healthy control individuals. The data is provided in nifti format^1^ only and each modality has been registered and resampled into a subject-specific co-ordinate frame using *NiftyReg* (Modat et al. 2010), an open-source registration software package available for download at: http://sourceforge.net/projects/niftyreg/. This is a registration and resampling routine based upon using cubic b-splines and normalized mutual information to realign imaging data. All resampling uses cubic resampling – although this can occasionally produce unphysical imaging values by under- or overshooting (such as negative MR signal values), this is offset by improved interpolation accuracy in high SNR regions. Registration of variable contrast data (either endogenous or exogenous in nature) remains a challenging task, and so with the exception of an affine T1-weighted to the non-diffusion weighted image, explicit registration is not carried out (Melbourne et al. 2007; Ben-Amitay et al. 2012). Registration might be necessary for datasets from real imaging populations and for well-defined regions of interest. Masks are provided which have been produced by intensity thresholding – the motivation in this work is to improve overall computation time but more advanced brain extraction techniques could be used if the user has them available. The simplicity of the multi-dimensional nifti format ensures easy file manipulation and coding within *NiftyFit*. Data includes diffusion weighted imaging (multi-shell data), T2 relaxometry (multi-echo and refocussed data), Arterial Spin Labelling (Pulsed ASL (PASL) and Pseudo-Continuous ASL (PCASL)), and inversion recovery data. T1-weighted imaging data is also provided as an anatomical reference. Parameter files are provided as plain text for diffusion b-values and b-vectors, flip angles and TE and TI times.

In the corresponding *NiftyFit* test data, cases 01-06 are volunteer data acquired using a 3T Siemens Trio (PASL, DWI, multi-echo T2, multi-inversion time T1). Subjects A-C are volunteer subjects acquired from a 3T Philips Achieva (PCASL and multiply-refocused multi-echo T2). We list the image contrast types that are available below:

- T1-weighted data is acquired using an MPRAGE acquisition at 1.1 *mm* isotropic and resampled to DWI space (Cases1-6, Siemens Trio) or PCASL space (CasesA-B, Philips Achieva),
- Inversion Recovery data is acquired at up to five inversion times between 500 and 5000 *ms* at 2.5 *mm* isotropic resolution (Cases1-6, Siemens Trio only),
- Multi-echo T2 relaxometry data is acquired at roughly 21 echo times, finely sampled between 19 and 50 *ms* and coarsely sampled from 50 to 150 *ms* at 2.5 *mm* isotropic resolution (Cases1-6, Siemens Trio only),
- Refocussed T2 relaxometry is acquired with an echo time of 12 *ms* for 32 echoes with a TR of 9 *s*. Resolution is 0.42 × 0.42 × 3*mm* (Case C, Philips Achieva only),
- Diffusion Weighted MRI is acquired on 3 shells, 8 directions at *b* = 300 *s.mm*^*−2*^, 32 directions at b = 700 *s.mm*^*−2*^ and 72 directions at b = 2000 *s.mm*^*−2*^ with 12 *b* = 0 volumes. Resolution is 2.5 *mm* isotropic (Cases1-6, Siemens Trio only),
- PCASL data is acquired for 30 control-label pairs using a 2D EPI read-out with Label Duration of 1650 *ms* and Post-Labelling delay of 1800 *ms*. Resolution is 2.5 × 2.5 × 6 *mm* (CasesA-B, Philips Achieva only),
- Pulsed ASL data is acquired for 5 averages using a 3D GraSE read-out with QUIPSSII pulse time and inversion time of 800 *ms* and 2000 *ms* respectively at 2.5 *mm* isotropic resolution, resampled into DWI space (Cases1-6, Siemens Trio only),

### Package Overview

*NiftyFit* is available for download as detailed in the information sharing statement. The package contains a selection of routines for model-fitting to different types of MRI data. Summary details of the fitting procedures are provided in the associated presentation file. The software currently fits models to four different types of MR data (executable in parenthesis):

- Arterial Spin Labeled MRI (fit_asl),
- Single and multi-component T1 relaxometry (fit_qt1),
- Single and multi-component T2 relaxometry (fit_qt2),
- Diffusion Weighted MRI (fit_dwi),
- Diffusion Tensor manipulation routines (fit_tools),
- Basic image maths and manipulation routines (fit_maths).

**Fig. 1:**
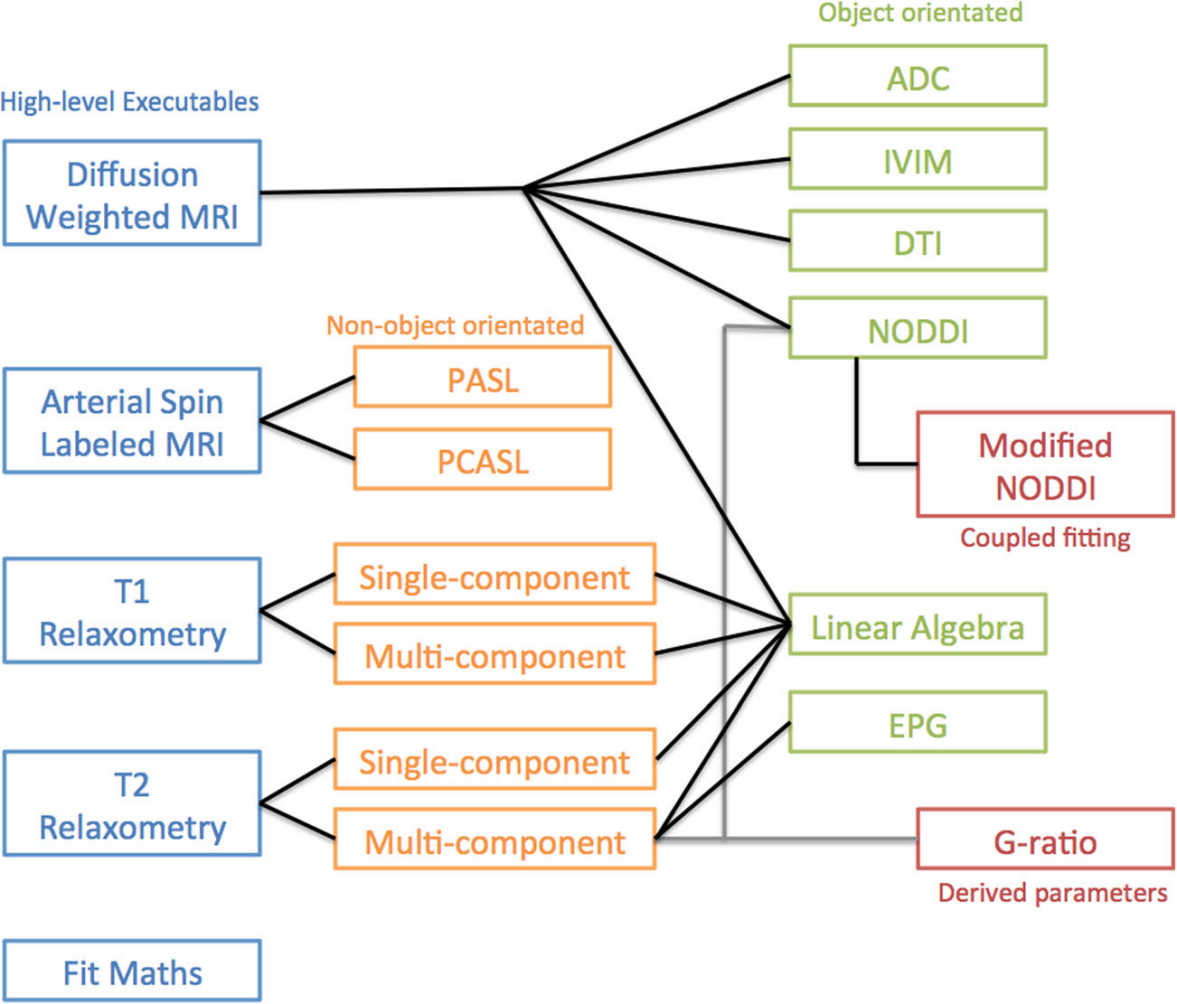
Schematic illustrating the organisation of the NiftyFit code and the inter-relationships between high-level executables, common libraries and derived parameter types

The software is organised as illustrated in Fig. 1. High-level executables allow fitting to mono-modal data types based around the themes above. These depend on non-object oriented code with the exception of DWI fitting routines that are object orientated. General fitting routines such as ordinary least squares are organised in a separate library so that they can be included within any new executable. A new fitting routine would, for example, have its own executable that would expect a particular data type and which would contain within it a bespoke fitting strategy that would call the lower level least-squares fitting routines explicit in the fitting library. If the user wishes to add a new generic fitting routine it can be added to the relevant library and the addition of a switch to the new fitting method in the main executable should be straightforward.

#### Underlying Input/Output Framework

*NiftyFit* provides some standardisation of its input and output across the individual modality fitting methods. Standard nifti image inputs are:

-source; the input data which expects a 4D nifti file organised with the dependent variable (e.g. time, echo time or diffusion weighting) along the fourth dimension,
-mask; a 3D mask file (optional, but recommended),
-init; a 4D initialisation parameter file in which the parameters are organised along the fourth dimension in the same order as the *NiftyFit* parameter output. These provide an initialisation for non-linear least squares. Currently parameter initialisation only applies to non-linear fitting routines,
-slice; select a single slice to run the model-fitting on,
-voxel; select a single voxel to run the model-fitting on,
- Input and output help text is displayed when running each command with no inputs.

Variables can be submitted either directly on the command line (e.g. -TEs in fit_qt2) or within a text file organised as a tab-delimited row vector (as in fit_dwi for -bvec and -bval), although this is variable if command line entries are not expected to be practical. Standard nifti image outputs are:

-mcmap; a multi-parameter map with parameters organised along the 4th dimension. This file contains all the information required to build a synthetic version of the data (when using the input experimental variables),
-resmap; a 3D volume of the per-voxel model-fit residuals,
-error; a 4D volume of the independent, identically distributed (I.I.D.) parameter errors organised with variances followed by covariances,
-syn; a 4D volume of data simulated from the fitted parameters and input variables.

#### Parameter Fitting Routines

All of the fitting methods in *NiftyFit* are built around common matrix solving techniques (these are available in Eigen http://eigen.tuxfamily.org). Solutions of the least squares problem are found using the pseudo-inverse of the system matrix and variations of this fitting routine: weighted Least Squares (LS); Non-Negative LS and non-linear least squares (NNLS), each make use of this framework.

##### Linear Least Squares

The LS problem seeks the solution to the equation *Ab* = *y* where in general *b* and *y* are best described by column vectors and *A* by a matrix. Making use of the matrix pseudo-inverse of *A*, (*A*^*T*^*A*)^− 1^*A*^*T*^, yields the least-squares solution (Eq. 1).

1 $$ b={\left({A}^TA\right)}^{-1}{A}^Ty $$

and,

2 $$ b={\left({A}^TWA\right)}^{-1}{A}^TWy $$

for weighted least squares, where the weight matrix is often diagonal and formed by the individual measurement precisions. This method is used for single-compartment model fitting for all imaging modalities: arterial spin labeled MRI, T1 and T2 relaxometry, diffusion MRI.

##### Non-linear Least Squares

In order to generalise this solving routine to non-linear problems, the assumption is often made that the solution is locally linear in the parameters and thus a LS-based routine can be contrived using the Jacobian matrix (Eq. 3).

3 $$ {A}_{ij}={\left[\frac{\delta f\left(x,b\right)}{\delta {b}_j}\right]}_i $$

The local residual at data point *i* of *n* is given by Eq. 4 for an arbitrary function *f* that is a function of the fixed parameter *x*_*i*_ and *n* parameters in *b*_*j*_ that we wish to estimate.

4 $$ \varDelta {y}_i={y}_i-f\left({x}_i,b\right) $$

The update equation is then given by the solution of Eq. 5,

5 $$ A\varDelta b=\varDelta y $$

6 $$ \varDelta b={\left({A}^TA\right)}^{-1}{A}^T\varDelta y $$

7 $$ {b}_{t+1}={b}_t+{\left({A}^TA\right)}^{-1}{A}^T\varDelta y $$

where the matrix *A* is the matrix of first-order derivatives of *f* by the parameters *b*. The update *Δb* is applied to the current parameter estimates *b* and the algorithm iterates until some convergence is reached: either the residual falls to a low level or a maximum number of iterations is reached. Using higher order gradient terms in the parameter update estimation is uncommon due to the computational cost of forming the Hessian matrix, although improvements in precision might be expected when close to a solution. Convergence for this routine is occasionally slow. To this end a heuristic update technique was devised by Levenberg and Marquardt (Levenberg 1944; Marquardt 1963), leading to the eponymous algorithm. In this case the update steps are given by:

8 $$ {b}_{t+1}={b}_t+{\left({A}^TA+\lambda \mathrm{diag}\left({A}^TA\right)\right)}^{-1}{A}^T\varDelta y $$

Where the value of *λ* is chosen to interpolate between gradient descent and Gauss-Newton nonlinear least squares. In *NiftyFit*, the value and rate, *r*, of decrease of *λ* are set empirically using the -lm option where *λ* is reduced after each iteration by *λ*_*t* + 1_ = *λ*_*t*_/*r*. Convergence of the least squares algorithm is guided by the total residual, Eq. 9,

9 $$ R={\displaystyle {\sum}_i^m{\left({y}_i-f\left({x}_i,b\right)\right)}^2} $$

and if this does not change appreciably the algorithm is stopped. Alternatively the algorithm runs for -maxit iterations. This method is used for model fitting of T1 and T2 relaxometry when a parameter initialisation is used, and for the non-linear models in the diffusion-weighted imaging section.

## Example Applications and Case Studies

### Example Applications

#### Single Inversion Time Arterial Spin Labeled MRI

Fitting of Cerebral Blood Flow (CBF) maps to ASL data follows the simple derived forms stated in the ISMRM Perfusion Study group recommendations on ASL acquisition for PASL (Eq. 13) and PCASL (Eq. 12) (Petersen et al. 2006; Alsop et al. 2014). Both of these models are derivations of the Buxton model (Buxton et al. 1998) under specific experimental conditions. The CBF value is quantified under a number of fairly liberal assumptions and presented in conventional units of ml/100 g/min. Acquisition proceeds by acquiring a number of pairs of control, *S*_*C*_, and label, *S*_*L*_, data. These pairs are averaged to generate single voxel values for the control and label signal. In addition a normalisation is needed and this can be estimated by, for instance, acquiring a proton-density weighted image, *S*_*PD*_, or acquiring a number of inversion or saturation recovery images at varying inversion time and fitting a T1 recovery curve (for more details see the next section on T1 relaxometry).

##### Pseudo-continuous ASL

ASL CBF maps can be estimated using pseudo-continuous ASL. In this case the relevant equation is:

10 $$ \mathrm{C}\mathrm{B}\mathrm{F}=\frac{6000\lambda }{2\alpha}\frac{e^{\mathrm{PLD}/\mathrm{T}{1}_{\mathrm{blood}}}}{\mathrm{T}{1}_{\mathrm{blood}}\left(1-{e}^{-\tau /\mathrm{T}{1}_{\mathrm{blood}}}\right)}\frac{\left({S}_C-{S}_L\right)}{S_{PD}}\left[\mathrm{ml}/100g/ \min \right] $$

where *λ* is the plasma/tissue partition coefficient, PLD the post-labelling delay between end of bolus and start of imaging, T1_blood_ the blood T1 value, *α* the labelling efficiency and *τ* the labelling pulse duration.

##### Pulsed ASL

ASL CBF maps can also be estimated for using pulsed ASL. In this case the relevant equation is:

11 $$ \mathrm{C}\mathrm{B}\mathrm{F}=\frac{6000\lambda }{2\alpha}\frac{e^{{\mathrm{TI}}_2/\mathrm{T}{1}_{\mathrm{blood}}}}{{\mathrm{TI}}_1}\frac{\left({S}_C-{S}_L\right)}{S_{PD}}\left[\mathrm{ml}/100g/ \min \right] $$

with TI_2_ and TI_1_ representing the times of the imaging inversion (similar to PLD for PCASL) and the time of the bolus clipping saturation pulse (QUIPSS-II) respectively.

##### Other fit_asl Features

*NiftyFit* for ASL contains a number of additional features: if multiple control and label pairs are submitted as the input, the method can estimate outliers by calculating a z-score on the intensity distributions in the images, this can be done on the raw image intensities, or based on the pairwise difference image (the -out option). This allows images corrupted by hardware artefacts to be filtered. However this method does not correct for motion artefacts that should be pre-corrected by a suitable strategy. Improved blood T1 values could be estimated using a function derived from population studies (Lu et al. 2004; Zhang et al. 2013).

In addition, partial volume correction options are available in 2D and 3D (Asllani et al. 2008). This method fits a least squares estimate to the CBF values within a local 2D or 3D kernel based on the assumption that the local grey and white matter CBFs are constant. This method should be used with caution since it pre-supposes an accurate grey and white matter segmentation and registration and does not provide quantitative results since the size of the partial volume kernel can be chosen arbitrarily. It may however provide an alternative CBF estimate that is in some way corrected for features such as grey matter atrophy. It should also be noted that this method is quite different in intention to the partial volume correction methods employed for Positron Emission Tomography (Thomas et al. 2011).

#### Single and Multi-component T1 Relaxometry

##### Inversion and Saturation Recovery

To estimate a single-voxel T1 value, *NiftyFit* uses non-linear LS to find the two parameters [*S*_0_, T1] in Eqs. 12 and 13, for either saturation recovery (S_sr_) or inversion recovery (S_ir_), given known values of the multiple inversion times TI. Estimation of these parameters is useful for instance, for quantification of Cerebral Blood Flow maps in ASL data. These are special cases of a more general inversion recovery equation.

12 $$ \begin{array}{rcl}{S}_{\mathrm{sr}}\left(\mathrm{T}\mathrm{I}\right)& =& {S}_0\left(1-{e}^{-\mathrm{T}\mathrm{I}/\mathrm{T}1}\right)\end{array} $$

13 $$ \begin{array}{rcl}{S}_{\mathrm{ir}}\left(\mathrm{T}\mathrm{I}\right)& =& {S}_0\left(1-2{e}^{-\mathrm{T}\mathrm{I}/\mathrm{T}1}+{e}^{-\mathrm{T}\mathrm{R}/\mathrm{T}1}\right)\end{array} $$

##### Other fit_qt1 Features

Multi-component T1 estimation can also be attempted using *NiftyFit*. In this case the goal is to estimate the volume fractions {*v*_*i*_} associated with a set of predefined T1s with ∑_*i*_ *v*_*i*_ = 1 (Equation 14 for saturation recovery and Eq. 15 for inversion recovery). The solution in this case is linear and proceeds using non-negative LS and will return the output volume fractions via the -comp and -mcmap output options. Necessary inputs in this case are the number of expected tissue components (-nc) followed by the pre-defined tissue T1s, given as either command line values (-T1s) or in a text file (-T1list).

14 $$ \begin{array}{rcl}{S}_{\mathrm{sr}}\left(v,\mathrm{T}\mathrm{I}\right)& =& {S}_0{\displaystyle {\sum}_i{v}_i\left(1-{e}^{-\mathrm{T}\mathrm{I}/\mathrm{T}1}\right)}\ \end{array} $$

15 $$ \begin{array}{rcl}{S}_{\mathrm{ir}}\left(v,\mathrm{T}\mathrm{I}\right)& =& {S}_0{\displaystyle {\sum}_i{v}_i\left(1-2{e}^{-\mathrm{T}\mathrm{I}/\mathrm{T}1}+{e}^{-\mathrm{T}\mathrm{R}/\mathrm{T}1}\right)}\end{array} $$

#### Single and Multi-component T2 Relaxometry

The T2 relaxometry in *NiftyFit* offers non-negative LS and non-linear LS routines for single- and multi-echo data. A single-component T2 estimate can be made by NLS fitting to Eq. 18 for a range of TEs in order to estimate [*S*_0_, T2] (Whittall et al. 1997).

16 $$ S\left(\mathrm{T}\mathrm{E}\right)={S}_0{e}^{-\mathrm{T}\mathrm{E}/\mathrm{T}2} $$

Similarly, multi-component T2 estimation can be carried out to estimate the volume fractions *v*_*i*_ associated with a set of predefined T2s: [{*v*_*i*_}, *S*_0_] where ∑_*i*_*v*(*i*) = 1. The solution is found either using non-negative LS, or if a component initialisation is provided, using non-linear least squares.

17 $$ S\left(\mathrm{T}\mathrm{E},\left\{\mathrm{T}2\right\}\right)={S}_0{\displaystyle {\sum}_i{v}_i{e}^{-\mathrm{T}\mathrm{E}/\mathrm{T}{2}_i}} $$

The experimental TEs can be entered in three ways, directly via the command line using the -TEs option, via a simple text file using the -TElist option, or, if the echo times are equally spaced by using the -TE option which contains the echo spacing (e.g. -TE 12).

##### The EPG Algorithm

If the T2 estimation experiment is carried out with repeated refocusing, as opposed to separate experiments with varying TE above (case16-qt2.nii.gz is an example dataset), then the signal modelling can become susceptible to errors due to B1 inhomogeneity. Multi-spin echo T2 decay generally assumes a train of perfect refocusing pulses that implies a perfectly homogenous B1 field (giving rise to Eq. 17). In practice this condition is not met as the scanner with the consequence that stimulated echoes are produced along the echo train. However, these may be modelled using the Extended Phase Graph (EPG) algorithm (Prasloski et al. 2012; Lebel and Wilman 2010) in such a way that the local refocusing angle, *α*, can be estimated by simulating the history of previous imperfect refocusing pulses Eq. 18). This algorithm simultaneously estimates the B1 inhomogeneity on a per-pixel basis (Fig. 11g).

18 $$ S\left(\mathrm{T}\mathrm{E},\left\{\mathrm{T}2\right\},\alpha \right)={S}_0{\displaystyle {\sum}_n{v}_i\mathrm{E}\mathrm{P}\mathrm{G}\left(\mathrm{T}\mathrm{E},\ \mathrm{T}{2}_i,\alpha \right)} $$

Although superficially complex (literally), the matrices that form part of the general EPG algorithm can be coded quite efficiently. This is because, although the general solution is complex, because the initial signal is pure real, the signal components oscillate between pure real and pure imaginary values, thus no complex number routines are actually required. T2 relaxometry using the EPG algorithm can be carried out using the -epg option and the resulting B1 map is output using the -b1map flag and the output is provided in radians, 0 ≤ *α* ≤ *π*.

#### Diffusion Weighted MRI

Diffusion Weighted MRI is now a staple of most MR imaging protocols and generates significant research output. The flexibility of the imaging technique means that it is highly amenable to new imaging challenges. Examples in this section of the paper are drawn from multi b-value, multi-direction data at b-values of [0, 300, 700, 2000]*s. mm*^− 2^ and some example images are shown in Fig. 11. The DWI routines within *NiftyFit* have been developed to allow the same model-fitting framework used in the other imaging modalities to be applied without bias. The incorporation of tensor and multi-compartment model fitting allows joint model-fitting to be carried out and we present some applications of how to do this in the following sections.

A number of methods are available for analysis of this data and some of these models are available in *NiftyFit*. In the most general case, fitting a mono-exponential isotropic decay is carried out using either log-linear or non-linear least squares to estimate the two parameters (a magnitude parameter *S*_*0*_ and rate parameter diffusion coefficient *d*) in Eq. 19 [*S*_0_, *d*]:

19 $$ S(b)={S}_0{e}^{-bd} $$

##### Diffusion Tensor Fitting

In the presence of multiple direction sampling (at least six directions, each direction described as a vector **r**), the Diffusion Tensor Imaging model (DTI) can be fitted (Le Bihan et al. 2001) (Eq. 20). The DTI model proceeds by LS fitting to the log of the signal. The resulting 3 × 3 symmetric matrix system, **D**, of diffusivities can then be diagonalised to estimate a principal diffusion direction (PDD) and set of diffusion eigenvalues from which parameters such as the mean diffusivity (MD) and the fractional anisotropy (FA) can be calculated. Using the *NiftyFit* -mcmap will produce a parameter map with the tensor elements followed by the *S*_0_ (signal magnitude) estimate: [**D**, *S*_0_]. This could be useful for instance within an iterative model-fitting driven registration scheme.

20 $$ S\left(b,\mathbf{r}\right)={S}_0{e}^{-b{\mathbf{r}}^T\mathbf{D}\mathbf{r}} $$

##### Neurite Orientation and Density Distribution Fitting

Multi-compartment model fitting of DWI can also be carried out with the Neurite Orientation and Density Distribution model (NODDI (Zhang et al. 2012)). The method as implemented here has a number of differences to the original algorithm proposed in Zhang et al. 2012. These include 1) the parameters are initialised using the diffusion tensor scheme, 2) the integration over the Watson distribution is carried out by a finite sampling scheme rather than analytically and 3) the noise model is Gaussian which empirically assumes a high SNR, which although is likely to provide a reasonable function for *χ*^2^ minimisation, may not give such good estimates for parameter precisions. The implementation of NODDI used in *NiftyFit* is designed for ease of adaptation and for code transparency.

The method uses a mixture of analytical derivatives for estimation of the volume fractions and PDD and a finite difference scheme for estimation of the orientation dispersion index *γ*. The diffusion model combines three signal components as a function of b-value, *b*, and gradient direction, **r**, from an isotropic space and a coupled intra- & extra- cellular space (Eqs. 21, 22, and 23). After constraining parallel (to the principal diffusion direction), *d*_‖_, and isotropic, *d*_*iso*_, diffusivities, four parameters remain to be estimated: an isotropic diffusion volume fraction, *v*_*iso*_; an intra-cellular volume fraction, *v*_*in*_ (the remaining extra-cellular volume fraction is given by *v*_*ex*_ = 1 − *v*_*in*_ − *v*_*iso*_); the oblateness of the fitted Watson distribution, 0 ≤ *γ* ≤ 1 (higher values tend towards a spheroid shape), used to infer white matter fibre dispersion, and the principal diffusion direction *μ*. Both *μ* and *γ* may be used to generate an extra-cellular component diffusion tensor D* for which there is an analytical equivalent of the expression: D*(*μ*, *γ*) = ∫_*Ω*_*f*(**n**|*μ*, *γ*)*D*(**n**)*d***n** when *f*(**n**) is a Watson distribution integrated over spherical space. In *NiftyFit*, representation of the PDD is in spherical polar coordinates, *p*(*θ*, *ϕ*), which allows this to be estimated simultaneously alongside the scalar parameters [*v*_*in*_, *v*_*iso*_, *γ*, *S*_0_, *θ*, *ϕ*]

21 $$ S\left(b,\mathbf{r}\right)={S}_0\left({v}_{in}{A}_{in}+{v}_{ex}{A}_{ex}+{v}_{iso}{e}^{-b{d}_o}\right) $$

22 $$ {A}_{in}={\displaystyle {\int}_{\varOmega }f\left(\mathbf{n}\right){e}^{-b{d}_{\left|\right|}\left(\mathbf{r}\cdot \mathbf{n}\right)}d\mathbf{n}} $$

23 $$ {A}_{ex}={e}^{-b\mathbf{r}{D}^{*}\mathbf{r}} $$

#### Example Application: Modified NODDI Fitting

The example described in this section demonstrates the possibility of combining traditionally separate model-fitting algorithms within a unified model. This can be used to enhance the fitting of existing parameters, or in the case of the following section, derive new model parameters from existing data.

The multi-compartment diffusion fitting routine above can be enhanced by the inclusion of T2 relaxometry data. In this case we give the algorithm additional information to fit the *v*_*iso*_ volume fraction. This is intrinsically acceptable (with caveats discussed below) because we expect the T2 relaxation time of the *v*_*iso*_ volume fraction to become long if it has a diffusivity of 3 × 10^−3^*mm*^2^*s*^−1^. We modify

24 $$ S\left(b,\mathbf{r}\right)={S}_0\left[{v}_{in}{A}_{in}+{v}_{ex}{A}_{ex}+{v}_{iso}{e}^{-b{d}_{iso}}\right] $$

To become

25 $$ S\left(b,\mathbf{r},\mathrm{T}\mathrm{E}\right)={S}_0\left[\left({v}_{in}{A}_{in}+{v}_{ex}{A}_{ex}\right){e}^{-\mathrm{T}\mathrm{E}/\mathrm{T}{2}_{wm}}+{v}_{iso}{e}^{-b{d}_{iso}}{e}^{-\mathrm{T}\mathrm{E}/\mathrm{T}{2}_{iso}}\right] $$

and we simplify this analysis by only varying the TE of the b0 images, in which case the equation simplifies in the absence of diffusion-weighting to become the two-component T2 relaxometry fit discussed in the T2 relaxometry section with fixed T2 values of T2_wm_ and T2_iso_. With Eq. 25 it is possible to see how the multi-compartment diffusion signal overlaps with a simplified multi-component T2 model (Melbourne et al. 2015).

#### Example Application: g-Ratio Estimation in Adult Controls

Combined fitting routines can be used to estimate novel imaging biomarkers as in Melbourne et al. 2014a. This section will recreate this analysis of the g-ratio as an example of using *NiftyFit* for multi-modal multi-parametric model-fitting. The g-ratio can be measured directly in vitro and more recently can be estimated as an emergent bulk property on MRI. The g-ratio is an interesting number as it relates to axonal conduction velocity and electrostatic energetic efficiency and it represents the ratio of internal axonal diameter to the total nerve diameter (axon+myelin) (Chomiak and Hu 2009).

**Fig. 2:**
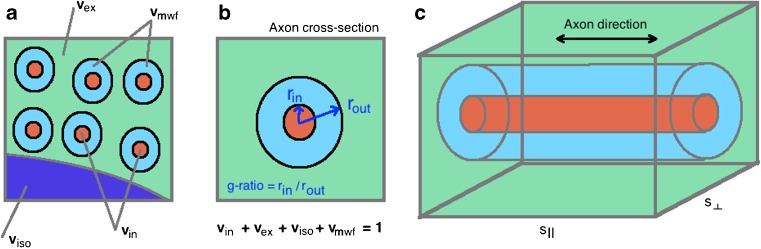
Illustration of emergent g-ratio estimation in MRI. **a** uniform parallel axons in cross-section demonstrating intra-axonal, myelin, CSF and extra-axonal spaces. **b** equivalent single axon model of multiple identical axons. **c** 3D volume sketch of 3D axon. A g-ratio may be measured in this instance from knowledge of the intra-axonal and myelin spaces (see text)

We start by imagining a set *n* of parallel axons (see Fig. 2). These axons are long cylinders with an internal axon radius of *r*_*in*_ and an external myelin+axonal radius of *r*_*out*_. Using the cylindrical geometry, the intra-axonal space is given by *v*_*in*_ ′ = *n*2*πr*_*in*_^2^*s*_||_ and the myelin volume by *v*_*mwf*_ = *n*2*π*(*r*_*out*_^2^ − *r*_*in*_^2^)*s*_||_ where *s*_||_ is a fixed axonal length. Simply taking the ratio of *v*_*mwf*_/*v*_*in*_ ′ yields an expression for the g-ratio, *Γ* (Equation 26) in terms of the myelin volume *v*_*mwf*_ and the intra-axonal volume *v*_*in*_ ′.

26 $$ \varGamma ={\left(\frac{v_{mwf}}{v_{in}\prime }+1\right)}^{-\frac{1}{2}} $$

Using only DWI or multi-component relaxometry is insufficient to estimate both *v*_*in*_ ′ and *v*_*mwf*_. To reconcile these two modalities we make use of a four-compartment tissue model (Alexander et al. 2010).

27 $$ {S}_{total}={v}_{mwf}{S}_1+{v}_{in}^{\prime }{S}_2+{v}_{ex}{S}_3+{v}_{iso}{S}_4 $$

The last three compartments of Eq. 27 are measurable using a multi-compartment diffusion model (Zhang et al. 2012). The model allows for the estimation of the signal from multiple compartments, specifically the intra-axonal volume fraction associated with highly directional structure, *v*_*in*_. The remaining model compartment for *S*_1_ describes signal associated, in white matter, primarily with myelin and can be estimated by T2 relaxometry. Finally, because the diffusion signal model contains no signal from *S*_1_ it is necessary to multiply the estimates of *v*_*in*_, *v*_*ex*_ and *v*_*iso*_ from the diffusion measurement by (1 − *v*_*mwf*_) and hence, *v*_*in*_ ′ = *v*_*in*_(1 − *v*_*mwf*_).

Estimation of the g-ratio can be carried out using *NiftyFit* as either a two-step process or a single step joint optimisation.

### Case Studies

Figures generated by the algorithms described in the preceding section are presented here for PCASL, Pulsed ASL, T1 and T2 relaxometry and DWI. Results from the combined fitting routines for the two applications described above are also shown. When included, processing speed data refer to the results of calculations performed on an Intel 3.5 Ghz i7 Mac with 32Gb DDR RAM.

#### Single Inversion Time Arterial Spin Labeled MRI

**Fig. 3:**
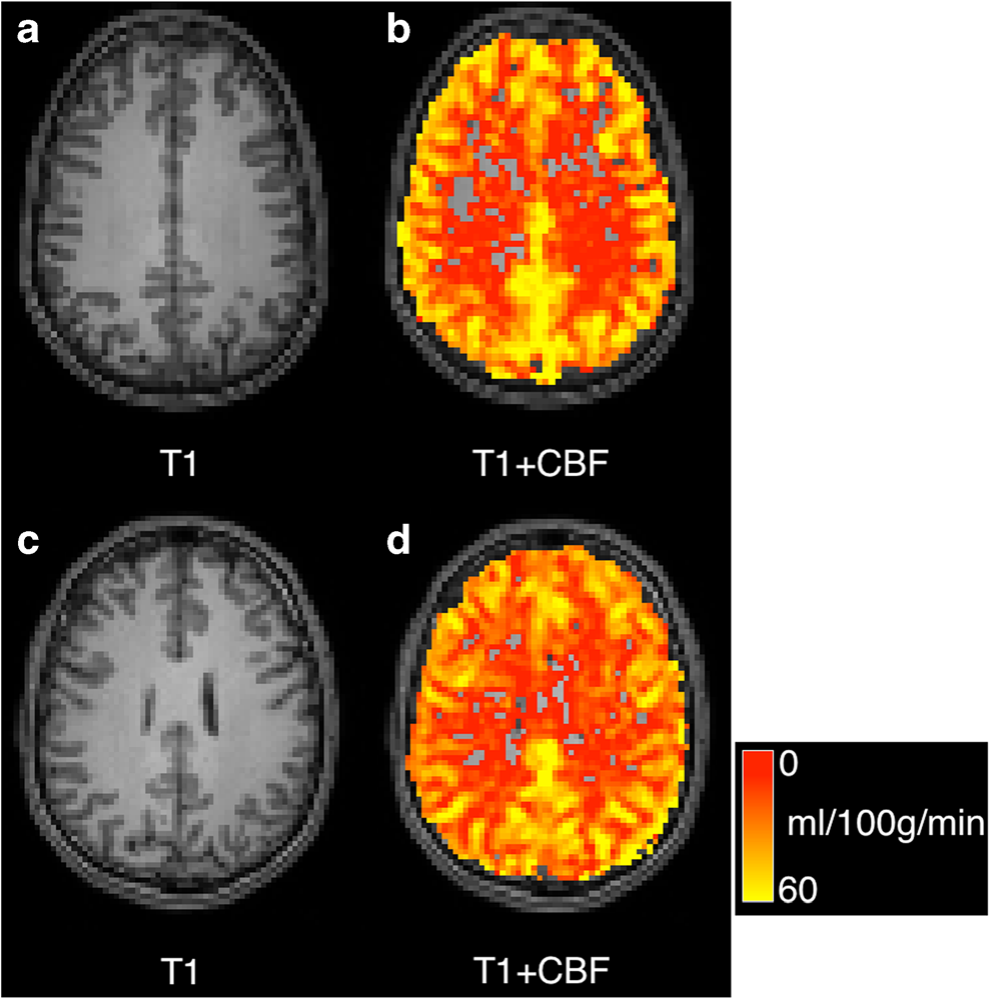
PCASL CBF images for case A (*left*) and case B (*right*). **a** T1-weighted image, **b** T1 weighted image overlaid with CBF map, **c** T1-weighted image, **d** T1 weighted image overlaid with CBF map. Missing voxels are thresholded to a value of zero, negative values are possible in regions of low perfusion, although they are likely to be the result of noise and motion

For PCASL, using the command:
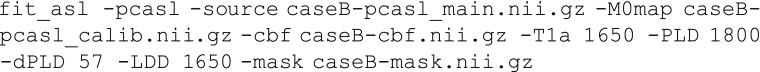


generates the images in Fig. 3 drawn from the estimated CBF map. Briefly, the major parameters entered are the blood T1 value, the post labelling delay (PLD), the slice-wise delay (-dPLD using 2D EPI) and the labelling pulse duration (all assumed to be in units of *ms*).

Using the following commands will generate CBF maps for PASL data where briefly, the major parameters entered are the blood T1 value, and the two labelling times for the first (labelling) and second inversion (imaging). In this case the images are acquired using 3D GraSE so there is no slicewise delay time.
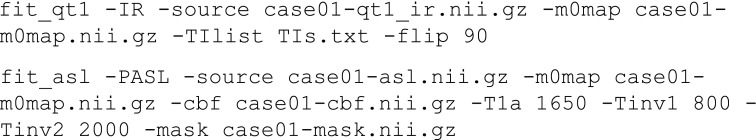


**Fig. 4:**
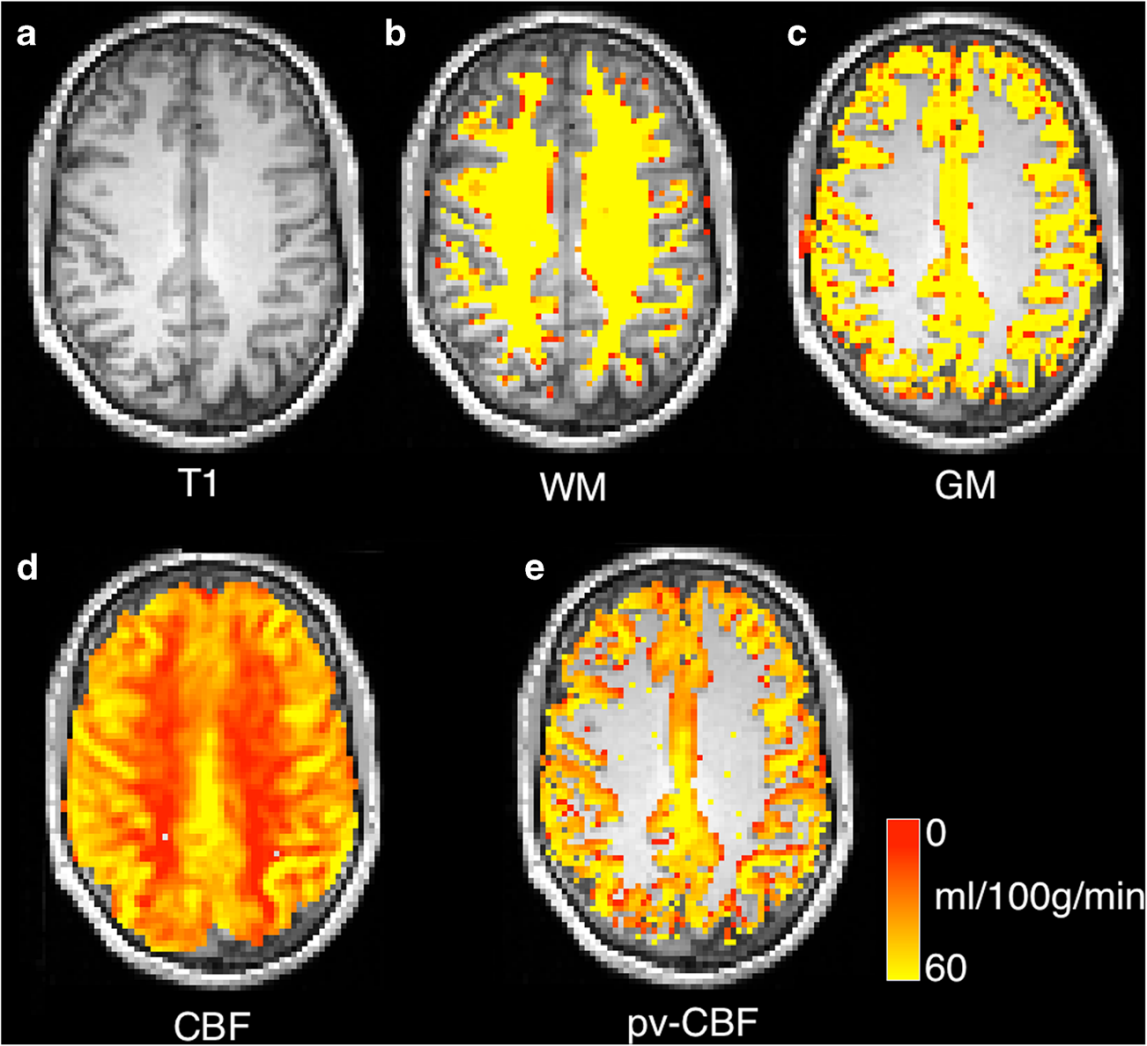
PASL CBF images for case01 with different partial-volume correction schemes for **a** anatomical image, **b** white matter segmentation, **c** grey matter segmentation, **d** CBF map generated using a separate M0 map estimation (see T1 relaxometry section) **e** partial volume correction using (Asllani et al. 2008) in 2d with a 3 × 3 kernel. All CBF maps overlaid on T1- weighted image

Figure 4 shows how the results of a tissue class segmentation may be used to carry out partial volume correction in PASL data.

Average runtimes for ASL fitting are typically less than 1 s.

#### Single Component T1 Relaxometry

**Fig. 5:**
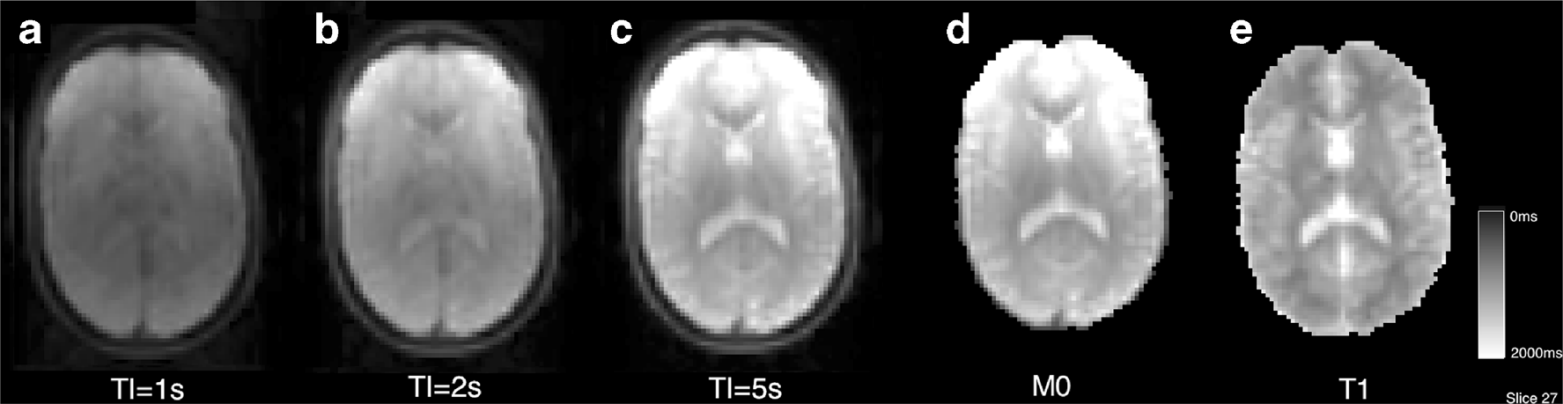
Saturation recovery T1 map generation (case02, slice 28)


The command below generates both a T1 estimate and an M0 map. The M0 estimate can be used to normalise an ASL CBF map as described above. An example of fitting is shown in Fig. 5 for three-timepoint saturation recovery.

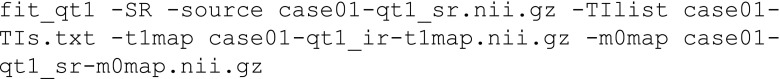


Average runtime across the six datasets for single-component T2 is 30 ± 5 *s*.

#### Single and Multi-component T2 Relaxometry

**Fig. 6:**
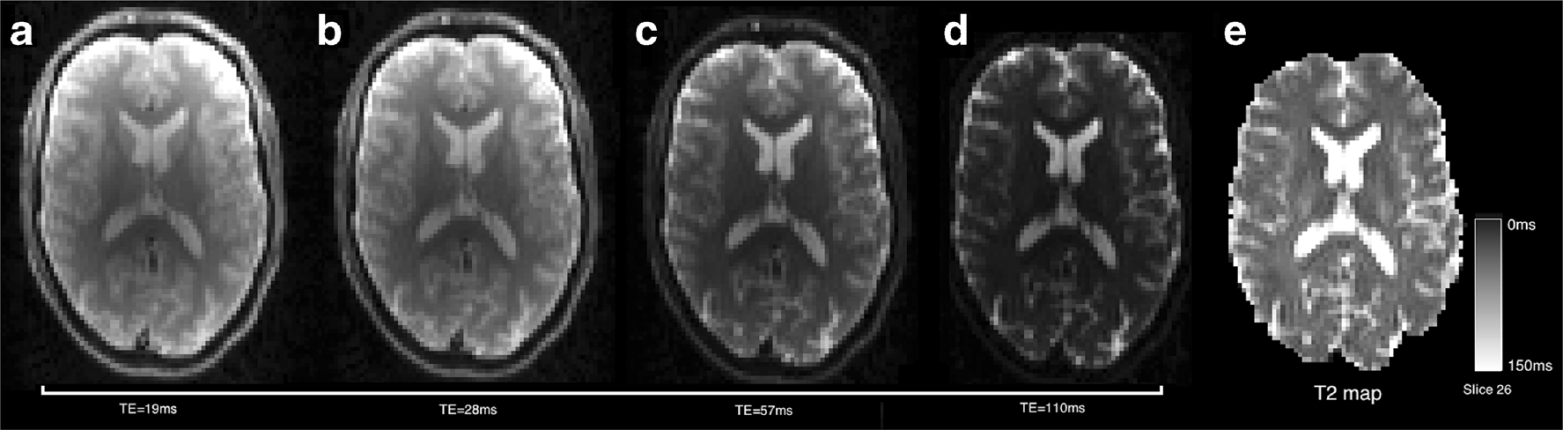
Example multi-echo T2-weighted acquisitions with four different echo times (**a**–**d**) and an estimated T2 map (**e**)

T2 relaxometry can be carried out with the following command to estimate a single-component T2 map,
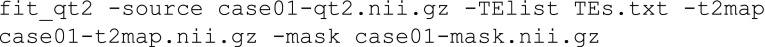


Figure 6 shows the results obtained by the above command. Echos at four times are shown, although the T2 map is fitted to all 19 echoes. Average runtime across the six datasets is 2 ± 1 *s*.

The single component estimated T2_1_ should be approximately equal to the grand mean T2, $\overline{\mathrm{T}2}={\displaystyle {\sum}_iv(i)\mathrm{T}{2}_i}$ when using a command such as:
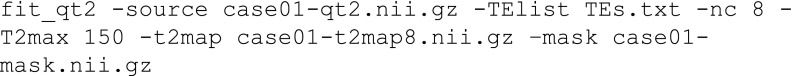


Multi-component T2 estimation can be carried out to produce a multi-component map. The mcmap.nii.gz output contains the volume fractions and the baseline signal magnitude estimate *S*_0_. The following command generates an output that broadly speaking contains two dominant volumes, a tissue component at about 50 *ms* and a fluid component at 150 *ms*, this maximum T2 relaxation time is perhaps best chosen by taking a regional CSF average from the single component fit.
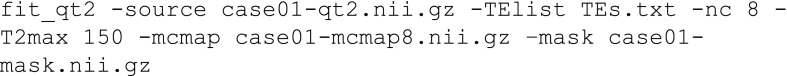


**Fig. 7:**
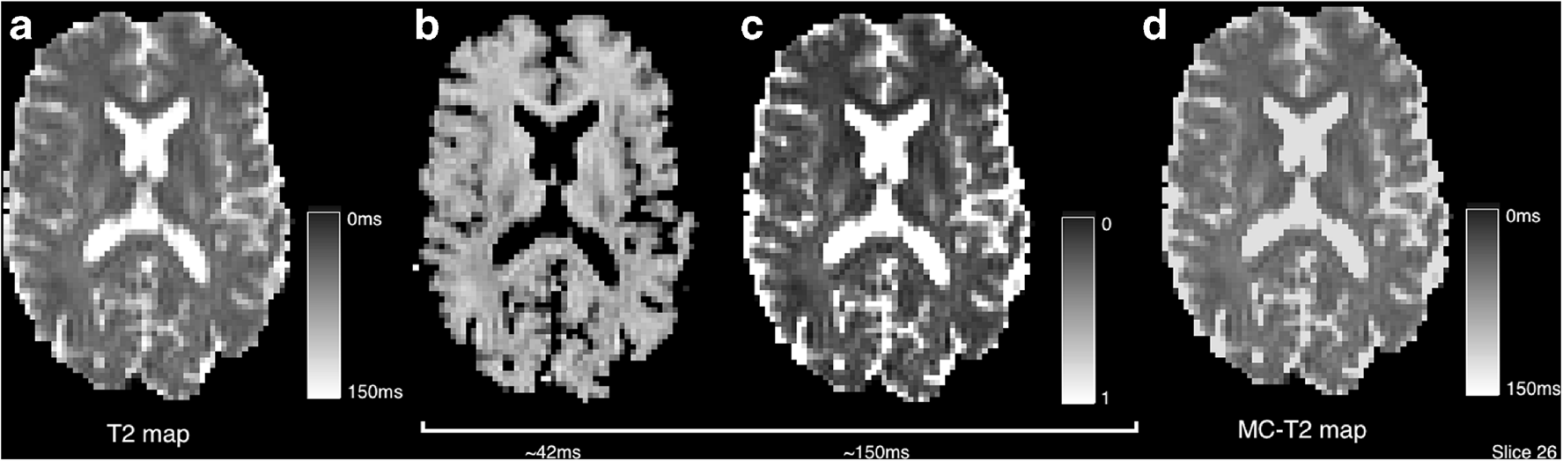
Comparison of single and multi-exponential T2 fitting. Two substantial components are found at approximately 42 *ms* and 150 *ms* and combined (**d**) these approximate the single-component T2 (**a**)

Figure 7 demonstrates the equivalence of the result when attempting to fit a multi-component exponential fit to this data. The NNLS algorithm generates a sparse solution of which there are two substantial components (shown) broadly separating tissue and non-tissue classes. Average runtime across the six datasets for estimation of eight components is 3 ± 1 *s*.

**Fig. 8:**
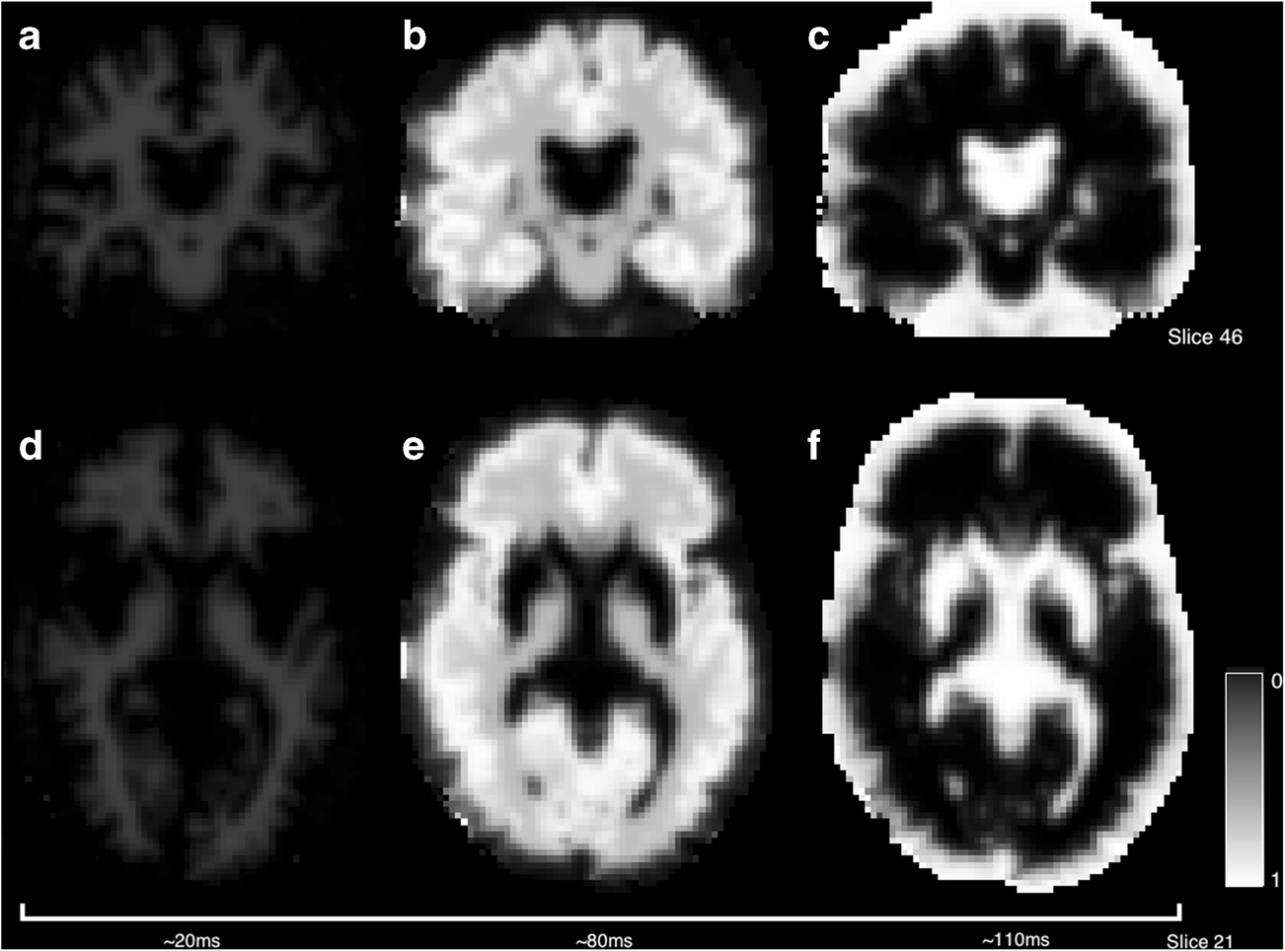
Example priors used to constrain a three-exponential fit to the data to attempt to extract an estimate of the myelin water fraction

**Fig. 9:**
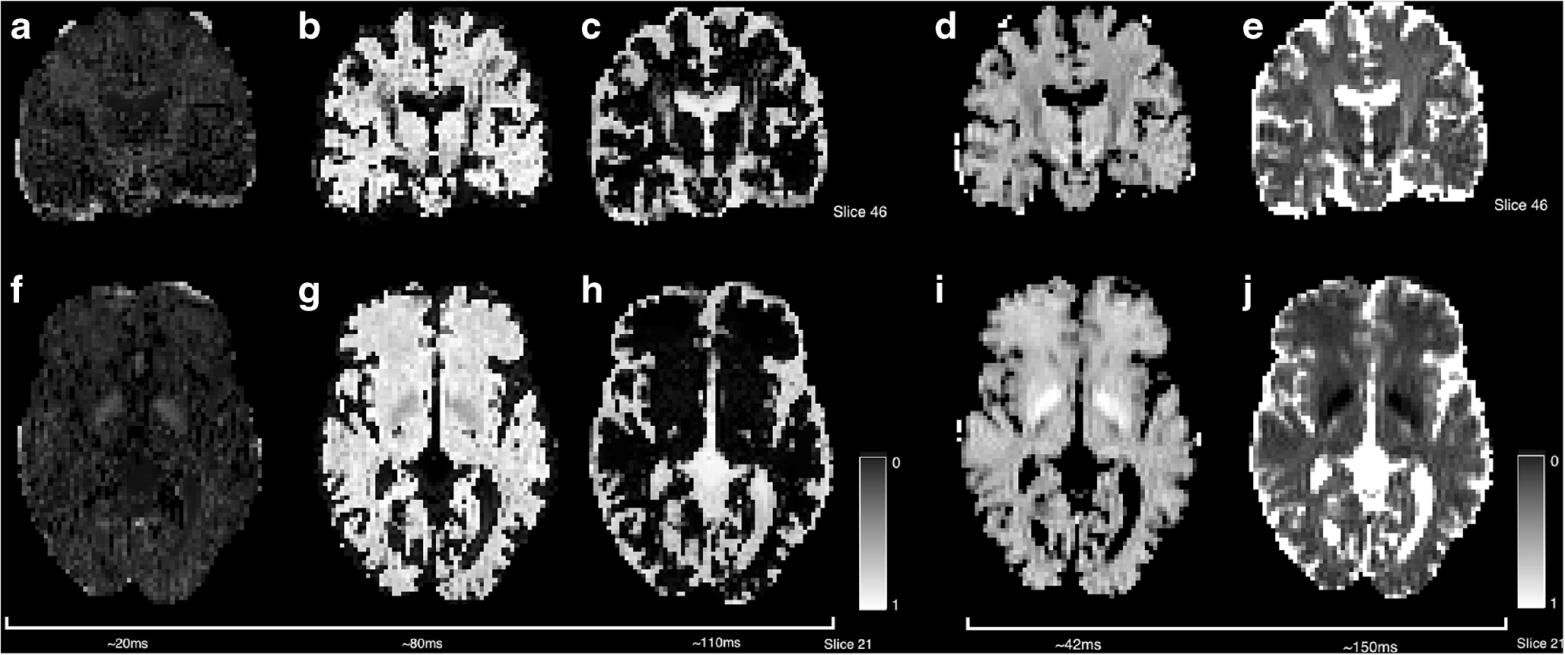
Using the priors in Fig. 7 allows estimation of a short T2 component (**a**, **f**) which may be used to approximate the myelin water fraction

Estimation of more than two-components from this data is difficult due to the range of T2s used. In this case a tissue prior might provide a suitable mechanism of constraining the fit with known anatomical information (Melbourne et al. 2013). Here we can use a prior derived from a segmentation (see Fig. 8) that provides voxelwise prior estimates for an additional volume fraction, namely the short T2 myelin water fraction (see Fig. 9). The myelin water fraction is defined as the sum of the volume fractions for T2’s less than 50 *ms* (this threshold can be set within *NiftyFit* using the -mwfthreshold option): $\mathrm{M}\mathrm{W}\mathrm{F}={\displaystyle {\sum}_{i=0}^{T{2}_i<50 ms}{v}_i}$.

The roundabout terminology’Myelin Water Fraction’ refers to the fact that this is not a direct measurement of myelin, but more a measurement of water that is presumed to have interacted with the myelin space over the course of the experiment, and thus have experienced T2 shortening. There is evidence that the MWF is linearly related to the myelin content in regions of white matter (Laule et al. 2006). Fitting a MWF proceeds using the following command:
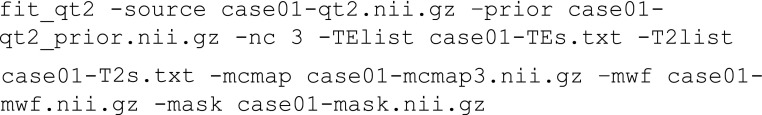


Average runtime across the six datasets when using an initialization is 25 ± 4 *s*.

**Fig. 10:**
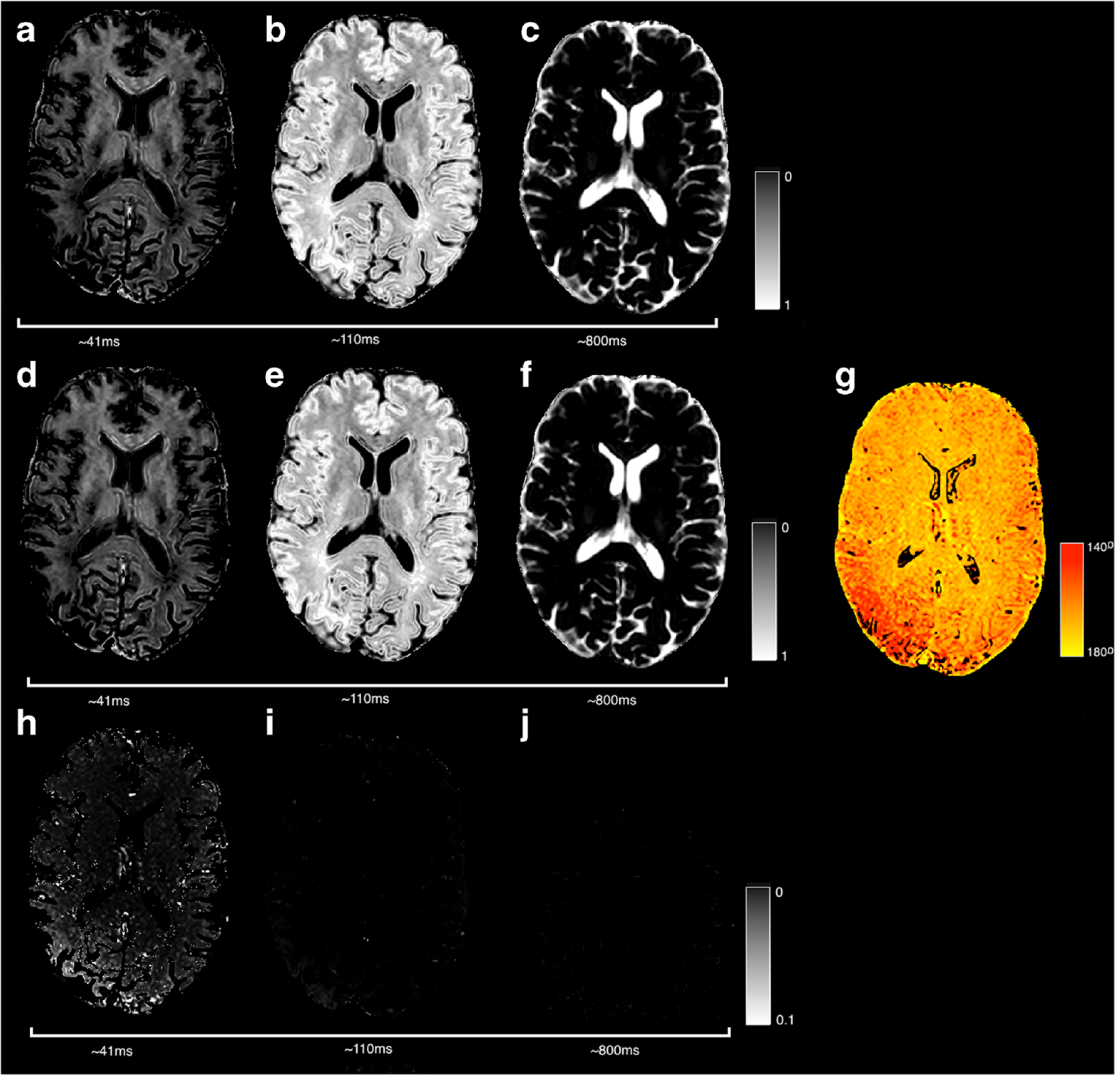
Comparison of a multi-compartment fit with (*top row*) and without (*middle row*) the EPG algorithm (case C). Differences (*bottom row*) are of note in the posterior right section of the short-component image which has higher component intensity when using the EPG algorithm. This region corresponds to a region of B1 inhomogeneity estimated by the EPG algorithm and displayed in G

Figure 10 provides an example of using the EPG algorithm to simultaneously correct for B1 field inhomogeneity during the multi-compartment T2 fitting. The influence of B1 inhomogeneity in repeatedly-refocused data is most clearly seen in the short T2 component. Runtime when using the EPG algorithm is 199 s, compared to 27 s when using the standard multi-exponential algorithm for a three-component fit.

#### Diffusion Weighted Imaging

**Fig. 11:**
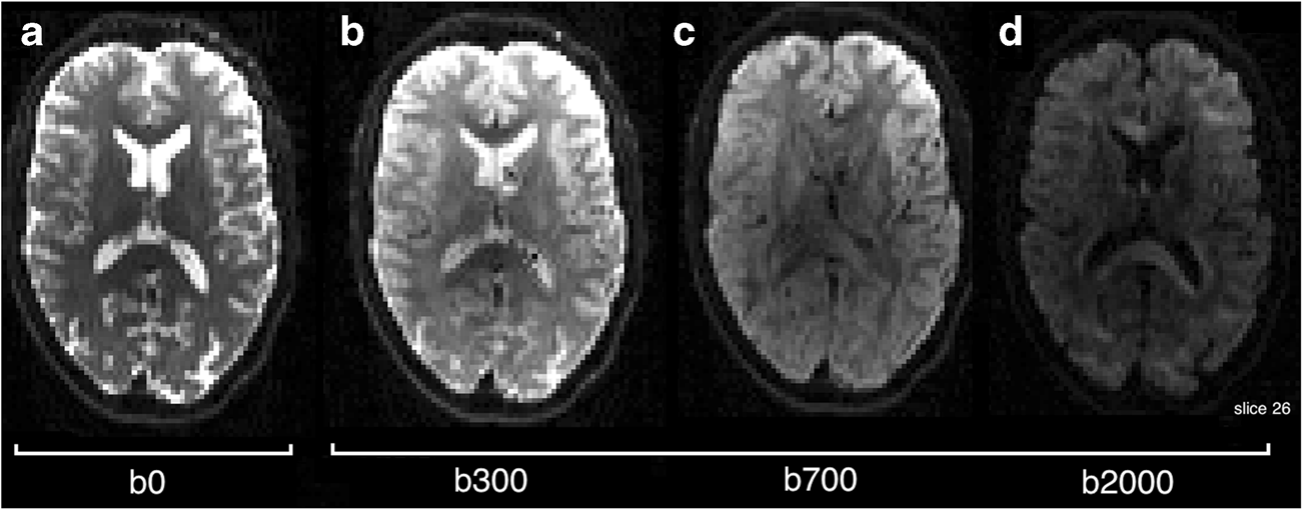
Example diffusion weighted images at b values of 0, 300, 700 and 2000 *s. mm* ^− 2^. Note the non-zero weighted images are scaled slightly differently to the b0 image and there is an arbitrary diffusion direction associated with each image

**Fig. 12:**
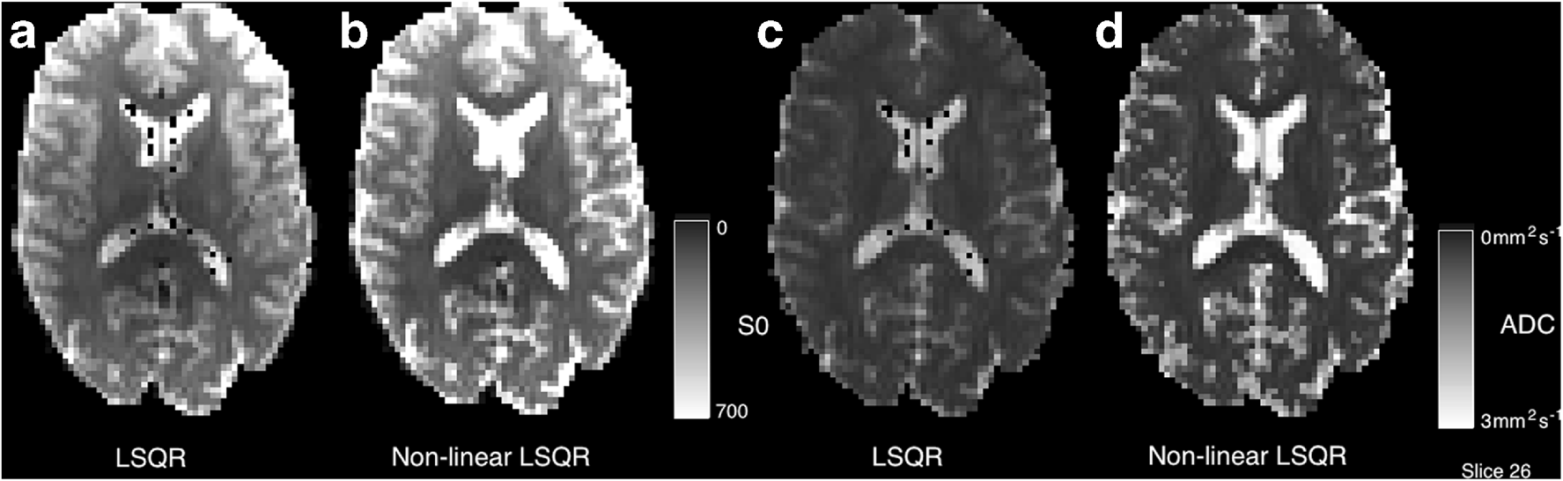
Comparison of log-linear and non-linear least squares fitting of the two parameter model in Eq. *19*. Note the dark voxels in the lateral ventricles are regions in which the least squares fit has failed in regions of low SNR


Data in this section consists of several b-values and repeated instances at b values of [300, 700, 2000]*s. mm*^− 2^ (Fig. 11). Of particular note is the higher diffusivity found by the non-linear least squares algorithm in regions of CSF partial volume (Fig. 12). Differences in these regions are driven by the log-transformation on the signal and noise properties, particularly at high b-value where the Gaussian noise model breaks down.

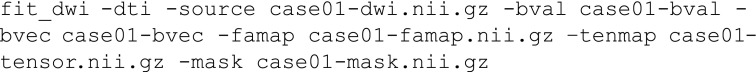


**Fig. 13:**
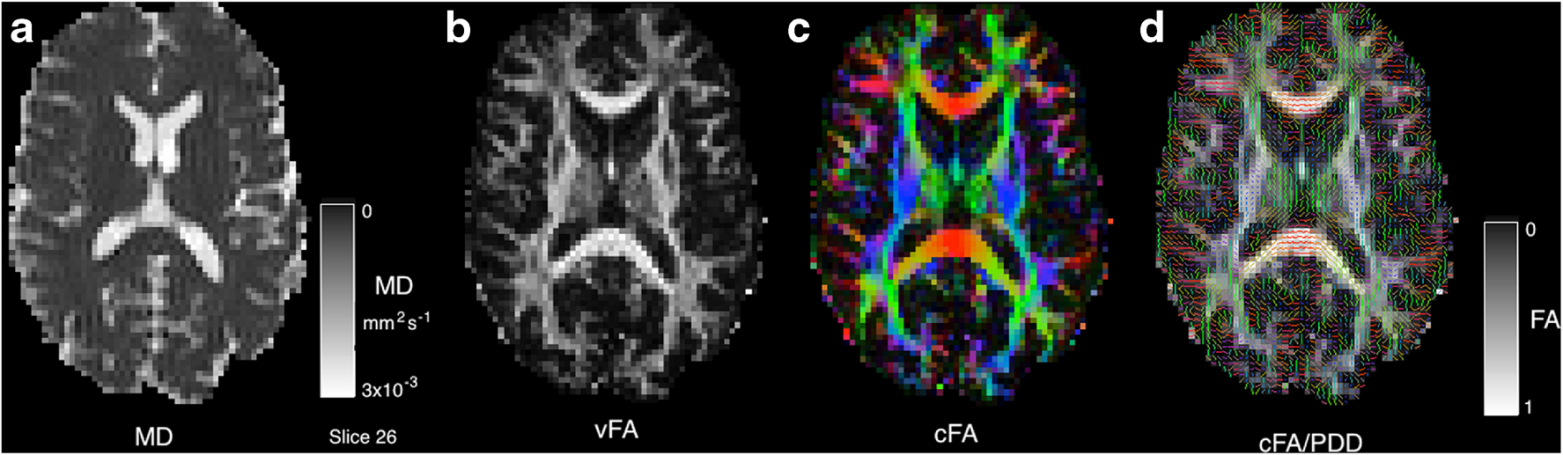
Diffusion Tensor fitting to estimate the mean diffusivity (MD), the fractional anisotropy (FA) and the Principle Diffusion Direction (PDD - overlaid as an unsigned vector)

Example fitting of diffusion tensor data is shown in Fig. 13 where we use the eigenvalues of the diffusion matrix **D** to form the mean diffusivity (the average of the eigenvalues, note this is subtly different to the diffusivity estimated in Eq. 19). The fractional anisotropy which represents the normalised average deviation of the eigenvalues from this mean value and the Principle Diffusion Direction (PDD) defined in the direction of the first eigenvector. Average runtime across the six datasets for DTI estimation is 4 ± 2 *s*.

**Fig. 14:**
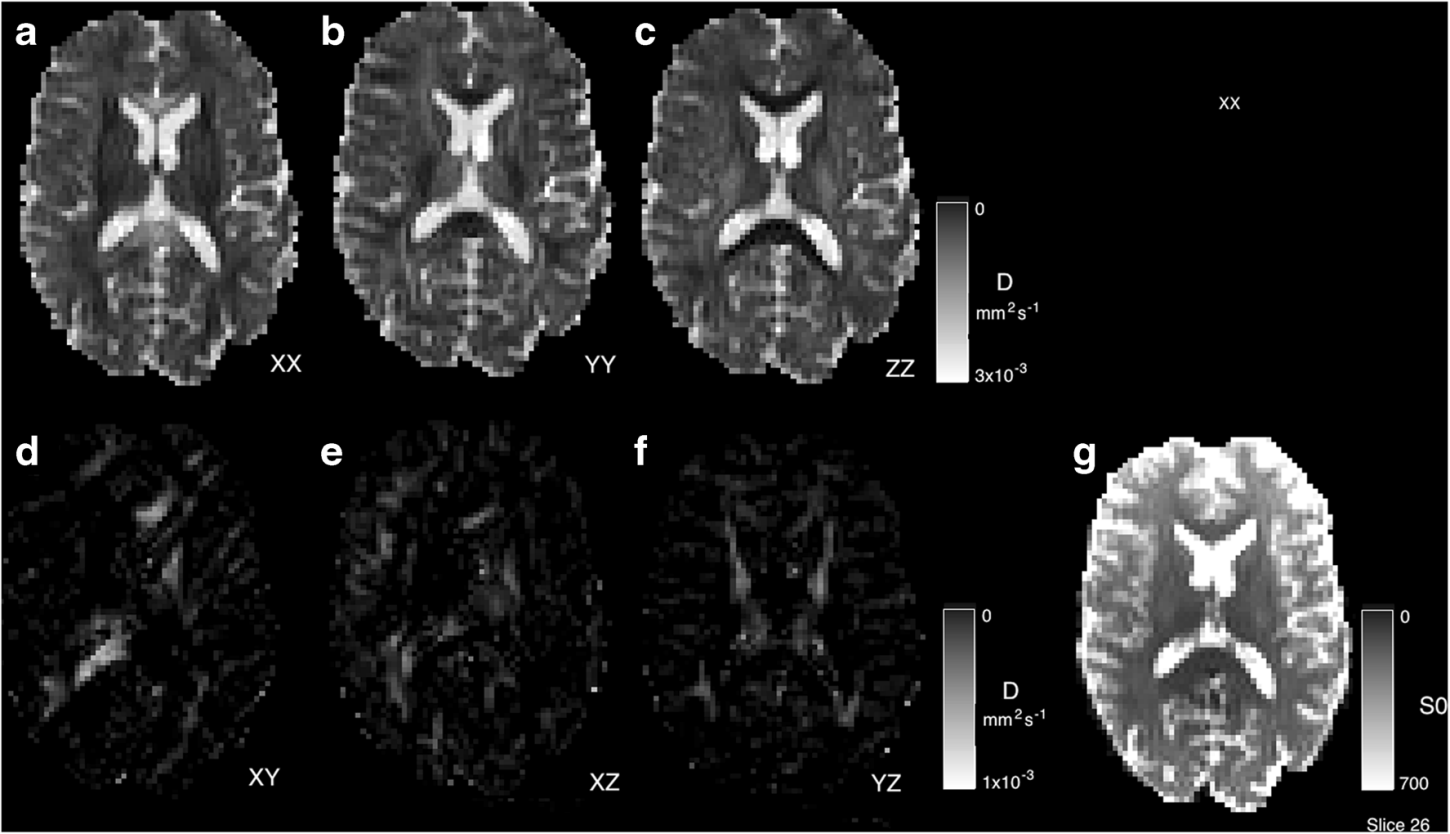
Fitted diffusion tensor components and initial signal level *S*_0_

The individual tensor components are illustrated in Fig. 14. This figure represents the output of the -mcmap option for DTI and enables the signal to be reconstructed using Eq. 20.

**Fig. 15:**
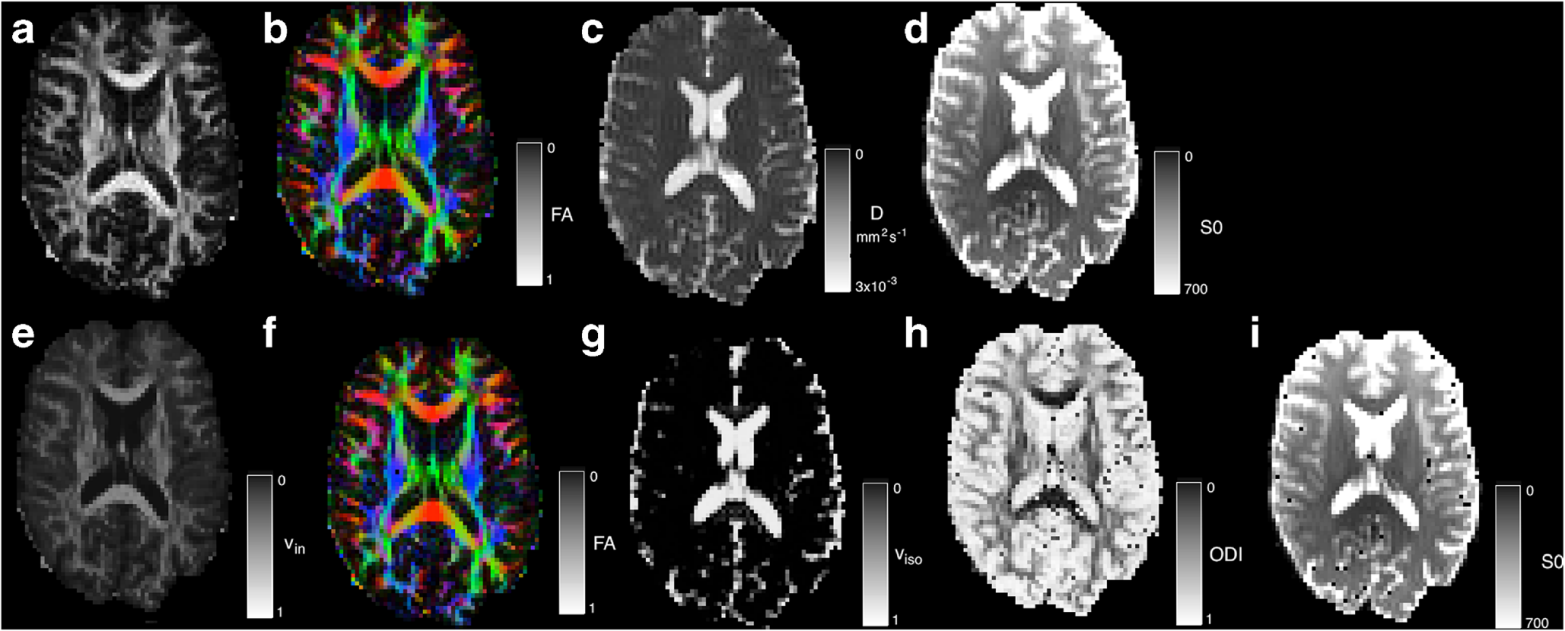
DWI parameter map comparison of DTI and NODDI parameters. **a**–**d**) represent DTI parameter estimates: **a** FA **b** colour-coded FA (*red* = ML, *green* = AP, *blue* = SI), **c**) the MD and **d**) S0. **e**–**i**) NODDI fitted parameters for **e** the intra-axonal volume fraction *v*_*in*_, **f** changes to the PDD, **g** the estimated isotropic volume fraction *v*_*iso*_, **h** the estimated tissue orientation dispersion index, ODI and **i** the S0


When using NODDI fitting, the -nod flag is used and output is assigned to the –mcmap output. An example of the output of this implementation is shown in Fig. 15. Occasionally the fitting procedure is sensitive to noise and results in erroneous fitting values. The effect of these voxels can be reduced by data or parameter smoothing. Average runtime across the six datasets for NODDI is 3609 ± 1357 *s*.

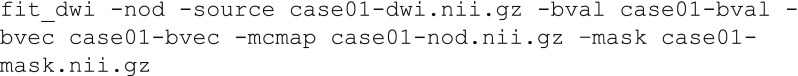


#### Modified Diffusion Weighted Imaging

The following command uses the additional variable -TE (with an additional text file) to modify the fitting.
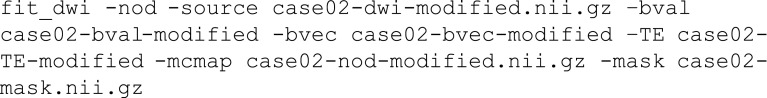


**Fig. 16:**
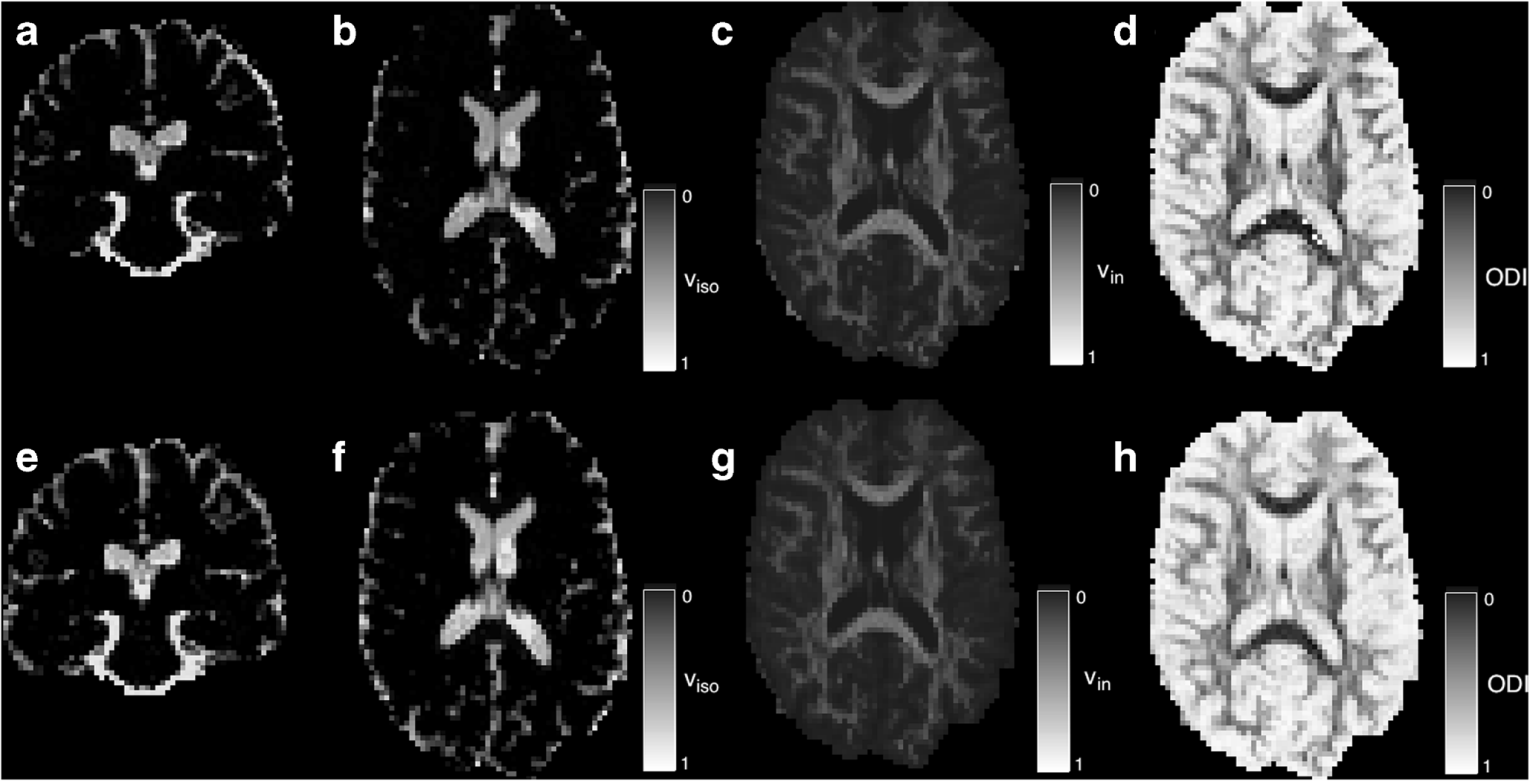
DWI NODDI parameter map comparison between standard and extended fitting. For isotropic volume fraction v_iso_, intra-axonal volume fraction v_in_ and the estimated tissue orientation dispersion index, ODI. Differences can be observed between regions of high *v*_*iso*_

Results are shown in Fig. 16. It should be noted that the interpretation of the difference in *v*_*iso*_ estimates is complicated slightly by the different treatment of perfusion effects. If these can be neglected, improved model-fitting performance can be achieved; conversely, if these effects cannot be neglected this methodology opens the door to more elaborate models of MR measurement (Melbourne et al. 2015).

#### g-ratio Estimation

##### Two-Step Estimation

Estimation of the g-ratio can be carried out using a two-step process:
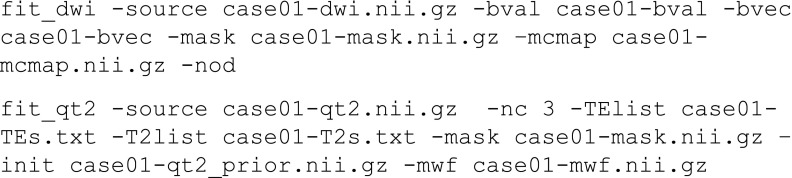


The g-ratio can be estimated using an external program to extract the first parameter estimate of case01-mcmap.nii.gz which represented the intra-axonal space *v*_*in*_ and the first parameter estimate of the T2 relaxometry result case01-t2comp.nii.gz which represents *v*_*mwf*_.
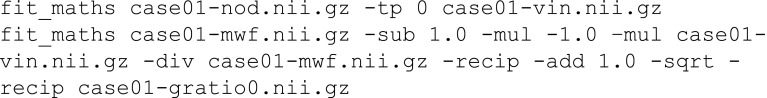


The voxelwise estimate $\varGamma ={\left({v}_{mwf}/\left(\left(1-{v}_{mwf}\right){v}_{in}\right)+1\right)}^{-\frac{1}{2}}$ is the estimated g-ratio at each voxel position. At the subject level, this estimate is quite noisy and the measurement may benefit from a region of interest-based approach.

##### Joint Model Fitting

The MRI data described above can be cast as a coupled optimisation since both models share common parameters, specifically *Γ* and *v*_*iso*_. We can define the independent parameters of the DWI signal model and the T2 relaxometry as *θ*_*a*_ and *θ*_*b*_ respectively with the shared parameters as *θ*_*ab*_ Thus, the signal model for the multi-component DWI can be summarised as *S*_*a*_ = *f*(*θ*_*a*_ = {*S*_*a*0_, *v*_*in*_, *γ*, *θ*, *ϕ*}, *θ*_*ab*_ = {*Γ*, *v*_*iso*_}) and the (adult) multi-compartment T2 sequence as *S*_*b*_ = *f*(*θ*_*b*_ = {*S*_*b*0_, *v*_*mwf*_, *v*_*tissue*_}, *θ*_*ab*_ = {*Γ*, *v*_*iso*_}). The application specific command below carries out joint fitting.
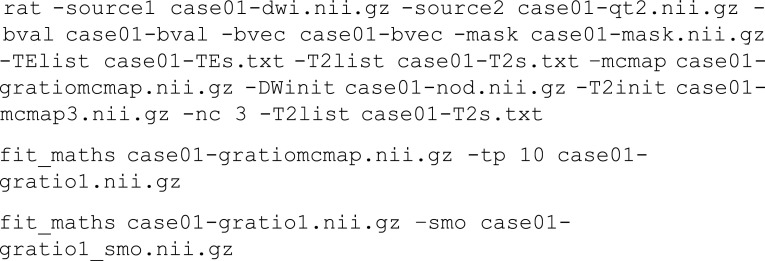


**Fig. 17:**
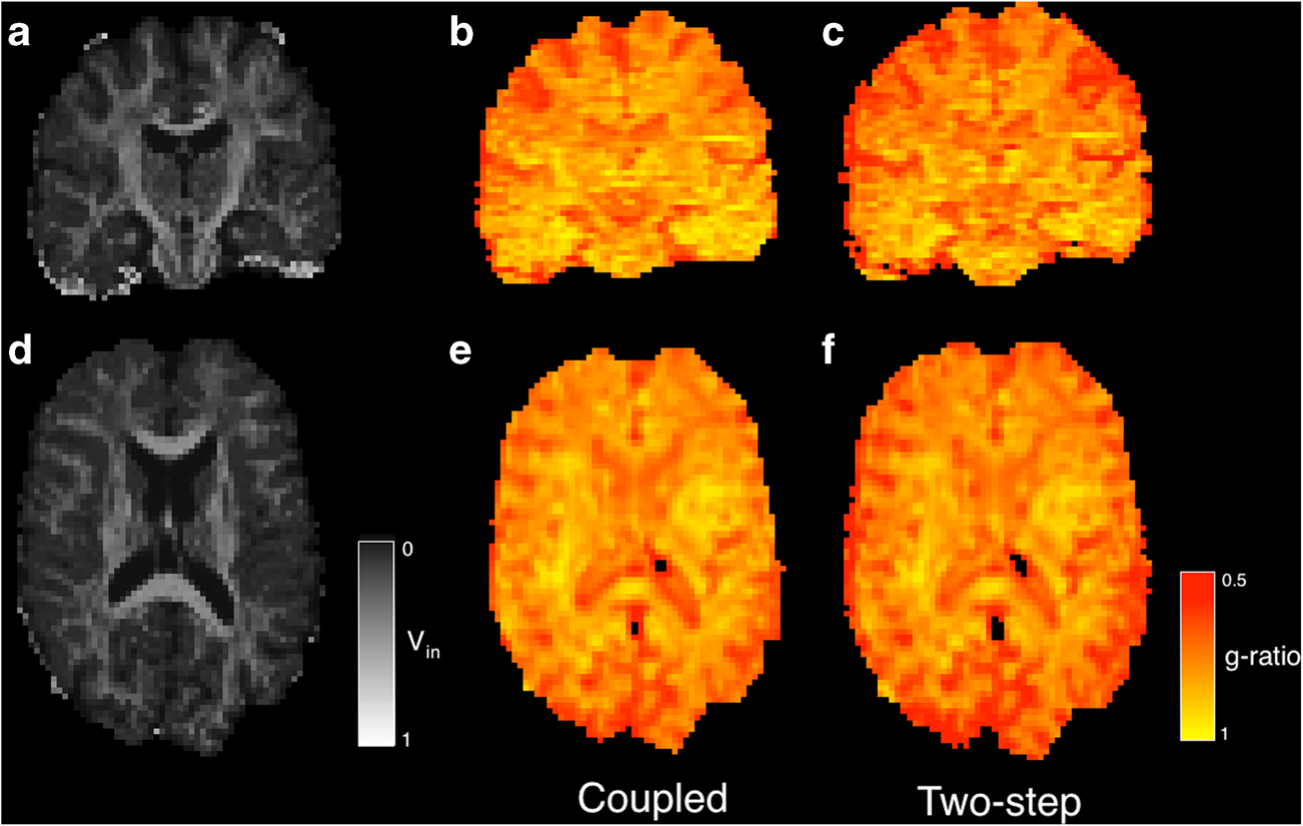
g-ratio estimation using two-step and joint estimation methods. **a**/**d**) joint v_in_ estimate. **b**/**e**) two-step g-ratio estimate and **c**/**f**) coupled estimate of g-ratio parameter. Images B/E/C/F smoothed using the fit_maths -smo option

Output of these two algorithms is provided in Fig. 17.

Since the estimates of *v*_*iso*_ made from diffusion imaging data and T2 relaxometry are made with different signal to noise ratios, the relative contributions of both types of data should be weighted. Specifically, the influence of the diffusion-weighted imaging on the *v*_*iso*_ estimates should be down-weighted relative to the T2 relaxometry estimate since they are likely to contribute more noise. As the first instance of a combined fitting routine, this example is illustrative. The method has a few limitations and these include the relatively noisy *v*_*mwf*_ estimate and a DWI model that considers only a single fibre direction. These represent future avenues for improvement and possible jumping off points to develop this software further and in a more specific fashion.

Table 1 contains white matter parameter values estimated from the DWI and T2 data from subjects 1–6. Myelin water fractions are estimated from T2 data, intra-axonal volume fractions from DWI data and g-ratio estimates calculated from independent DWI and T2 measurements or via coupled fitting. Standard FA and single component T2 measurements are also included. White matter values are provided using the white matter segmentations layers from the corresponding segmentations. The contribution of the coupled fit appears modest for these white matter average parameter values. The values for white matter g-ratio can be compared with direct histological measurements such as those in Stikov et al. 2015. Here the authors used slightly different techniques for estimating the axonal and myelin contributions, the authors found variation in the g-ratio along the length of the corpus callosum and state MRI values ranging between 0.63 in the genu and up to 0.79 in the callosal mid-body which match well to the corresponding histological measurements. Since the corpus callosum consists of highly packed fibres with a well-defined orientation, the values found in this region should be lower, on average, than the general white matter that has more varied orientation and different neurological optimisation requirements, both of which act to increase the g-ratio measurement. Excepting errors due to modeling error, image noise, and errors when associating histology with in vivo measurement, the values in Table 1 are within the plausible range, although the v_mwf_ estimates are consistently lower than in Stikov et al. 2015. An analysis of the propagation of error through the respective v_in_ and v_mwf_ estimates will help inform on the potential utility of g-ratio measurement over and above these mono-modal imaging modalities and it may be that a consistent measurement of the g-ratio is more valuable than an accurate one. It is also likely that the combined measurement will have a different sensitivity characteristics than the separate measurements of axonal and myelin density.

**Table 1:** Average white matter parameter estimates for cases 1–6 from DWI and T2 relaxometry

	Volume			Independent fitting			Coupled fitting		
Case	(mm^3^)	FA	T2 (ms)	V_in_	V_mwf_	g-ratio	V_in_	V_mwf_	g-ratio
1	495,583	0.423	71.808	0.514	0.141	0.864	0.526	0.176	0.887
2	422,227	0.439	70.021	0.498	0.137	0.869	0.509	0.134	0.868
3	390,378	0.431	70.712	0.518	0.147	0.862	0.530	0.143	0.855
4	433,189	0.413	70.115	0.516	0.159	0.848	0.530	0.155	0.841
5	464,354	0.409	66.101	0.502	0.162	0.845	0.515	0.158	0.841
6	431,296	0.351	69.848	0.470	0.163	0.835	0.482	0.159	0.831

FA and T2 are estimated from standard DTI and single-component relaxometry whilst v_in_, v_mwf_ are estimated from NODDI and multi-component relaxometry

## Discussion and Future Developments

This work has presented *NiftyFit* as a platform for development in multi-modal multi-parametric MR neuroimaging. Initial functionality has been demonstrated in ASL, DWI, and T1 and T2 relaxometry data. The method and results have been deliberately presented in a simple and pedagogic fashion to maximize the potential of the source code and data for educational and extensible purposes. The figures generated in this work are as reproducible as possible provided that the user has access to image display software. Future extensions are planned to include the Incoherent Vascular Incoherent Motion (IVIM, (Vos et al. 2015)) diffusion model, SPGR-based T1 relaxometry, BOLD imaging and pharmacokinetic modelling for Dynamic Contrast Enhanced MRI (Orton et al. 2008). Additionally, functionality for Bayesian fitting will be included based upon previous work (Chappell et al. 2009; Orton et al. 2014), although this must be used with caution in modalities when non-Gaussian noise becomes significant such as high-b-value DWI. Although all examples in this work are applied to neuroimaging, many of the techniques can be applied to imaging data from other regions of interest, for instance the liver or kidney. Early versions of this work have already supported publications by the authors, including (Melbourne et al. 2014a; Hamy et al. 2014) and (Melbourne et al. 2014b; Melbourne et al. 2015). Recent applications of sparse mathematics to model fitting (Daduccia et al. 2015) provide an alternative to non-linear and non-negative least squares and can be adapted to some of the other modalities described here. Some of the techniques developed in this work can be re-configured so that they are able to operate on graphical processing units. Software libraries already exist for GPU based linear algebra and the ability to incorporate these within NiftyFit and to run parallel operations on voxels from large datasets would lead to significant performance enhancement.

The intention with this release is to provide a simple, pedagogic code base that remains useful to future researchers, so that it may be modified, improved and extended, as the user requires. Key to this is the simultaneous release of software and imaging data with which to reproduce the figures in this work and allow the reader to explore the types of data intended for analysis. As a framework for future multi-modal multi-parametric model-fitting, this simplicity and unification of fitting routines is likely to offer much potential for future, as yet unanticipated, MR biomarker developments.

## Information Sharing Statement

Data and source code for *NiftyFit* (RRID:SCR_014301) are available at the following link: https://cmiclab.cs.ucl.ac.uk/CMIC/NiftyFit-Release.

## Footnotes

1 http://nifti.nimh.nih.gov/nifti-1
